# Obesity Accelerates Alzheimer-Related Pathology in *APOE4* but not *APOE3* Mice

**DOI:** 10.1523/ENEURO.0077-17.2017

**Published:** 2017-06-13

**Authors:** V. Alexandra Moser, Christian J. Pike

**Affiliations:** Neuroscience Graduate Program, Leonard Davis School of Gerontology, University of Southern California, Los Angeles, CA 90089

**Keywords:** Alzheimer’s disease, apolipoprotein E, β-amyloid, gliosis, obesity, transgenic

## Abstract

Alzheimer’s disease (AD) risk is modified by both genetic and environmental risk factors, which are believed to interact to cooperatively modify pathogenesis. Although numerous genetic and environmental risk factors for AD have been identified, relatively little is known about potential gene-environment interactions in regulating disease risk. The strongest genetic risk factor for late-onset AD is the ε4 allele of apolipoprotein E (*APOE*4). An important modifiable risk factor for AD is obesity, which has been shown to increase AD risk in humans and accelerate development of AD-related pathology in rodent models. Potential interactions between *APOE*4 and obesity are suggested by the literature but have not been thoroughly investigated. In the current study, we evaluated this relationship by studying the effects of diet-induced obesity (DIO) in the EFAD mouse model, which combines familial AD transgenes with human *APOE*3 or *APOE*4. Male E3FAD and E4FAD mice were maintained for 12 weeks on either a control diet or a Western diet high in saturated fat and sugars. We observed that metabolic outcomes of DIO were similar in E3FAD and E4FAD mice. Importantly, our data showed a significant interaction between diet and *APOE* genotype on AD-related outcomes in which Western diet was associated with robust increases in amyloid deposits, β-amyloid burden, and glial activation in E4FAD but not in E3FAD mice. These findings demonstrate an important gene-environment interaction in an AD mouse model that suggests that AD risk associated with obesity is strongly influenced by *APOE* genotype.

## Significance Statement

The ε4 allele of apolipoprotein E (*APOE*4) is the strongest genetic risk factor for Alzheimer’s disease (AD), but not all *APOE*4 carriers will develop the disease suggesting that *APOE* genotype interacts with other factors to modulate Alzheimer’s risk. Here, we show that diet-induced obesity (DIO) interacts with *APOE*4 genotype to increase Alzheimer’s-like pathology in an Alzheimer’s transgenic mouse model that contains human *APOE*3 versus *APOE*4 isoforms. Interestingly, mice with *APOE*3 do not show diet-induced increases in pathology, suggesting that the adverse effects of obesity on Alzheimer’s risk may be limited to *APOE*4 carriers. These findings identify an important gene-environment interaction that may have significant impact for understanding Alzheimer’s risk and etiology and promoting development of targeted therapeutic approaches that incorporate both obesity and *APOE* genotype.

## Introduction

Alzheimer’s disease (AD) is a progressive neurodegenerative disorder, the underlying causes of which are currently incompletely understood. Both genetic and environmental factors are important in determining individual risk for AD. The strongest genetic risk factor for late-onset AD is the ε4 allele of apolipoprotein E (*APOE*4; Strittmatter et al., 1993; Liu et al., 2013). In the United States, roughly 12% of the population carries the ε4 allele, but its frequency increases to ∼60% in AD patients (Rebeck et al., 1993). *APOE*4 not only increases risk, but also accelerates the age of onset of AD (Corder et al., 1993; van der Flier et al., 2011). However, since homozygous carriers of *APOE*4 have a ∼50% lifetime risk of AD, a significant number of *APOE*4 carriers never develop the disease (Genin et al., 2011). Thus, *APOE*4 likely interacts with other genetic and or environmental factors to drive AD risk.

A significant modifiable risk factor for dementia is obesity. Obesity has numerous adverse neural effects (Lee and Mattson, 2013) and increases the risk of dementia up to three-fold (Whitmer et al., 2008). Body mass index, a commonly used measure of obesity, has been shown to be associated with AD risk (Profenno et al., 2010) as well as with reduced brain volume in AD patients (Ho et al., 2010). Several studies indicate that obesity may be particularly problematic at midlife (Fitzpatrick et al., 2009; Profenno et al., 2010; Meng et al., 2014; Emmerzaal et al., 2015), suggesting that obesity contributes to the development of AD. Similar relationships have been observed in animal models. In particular, diet-induced obesity (DIO) accelerates AD-related pathology in mouse models of AD (Ho et al., 2004; Julien et al., 2010; Kohjima et al., 2010; Barron et al., 2013; Orr et al., 2014). Further, genetic models of obesity and type 2 diabetes exhibit features of AD-like neuropathology (Kim et al., 2009; Jung et al., 2013; Ramos-Rodriguez et al., 2013).

The extent to which *APOE*4 and obesity interact to regulate AD risk is unclear. Interestingly, *APOE*4 carriers can be more sensitive to metabolic consequences associated with obesity (de-Andrade et al., 2000; Kypreos et al., 2009; Niu et al., 2009; Atabek et al., 2012; Zarkesh et al., 2012; Guan et al., 2013). Although some studies do not report an *APOE*4 bias in obesity-associated AD risk (Profenno and Faraone, 2008; Luchsinger et al., 2012), others have found that AD risk is increased by obesity (Peila et al., 2002; Ghebranious et al., 2011) and diets high in calories and fatty acids (Luchsinger et al., 2002) only in *APOE*4 carriers. Though the human literature suggests a gene-environment interaction between *APOE* and obesity in regulating development of AD, this question has not been addressed in experimental models. To study these relationships, we used EFAD transgenic mice, which combine AD transgenes with targeted replacement of mouse *APOE* with human *APOE* (Youmans et al., 2012). We compared metabolic and AD-related effects of Western diet in male *APOE*3 (E3FAD) and *APOE*4 (E4FAD) mice. Here, we report that DIO increases amyloid pathology and gliosis almost exclusively in E4FAD mice. Our data reveal a gene-environment interaction between *APOE* genotype and obesity, suggesting that *APOE*4 carriers may be more susceptible to obesity associated increases in AD risk.

## Materials and Methods

### Animal procedures

A colony of EFAD mice, which are heterozygous for the 5xFAD transgenes and homozygous for human *APOE*3 or *APOE*4 (Youmans et al., 2012), were maintained at vivarium facilities at University of Southern California from breeder mice generously provided by Dr. Mary Jo LaDu (University of Illinois at Chicago). All animals were housed under a 12-hour light/dark cycle with lights on at 6 A.M. and *ad libitum* access to food and water. At three months of age, male E3FAD and E4FAD mice were randomized to dietary treatment groups (*N* = 7–11/group): control diet (10% fat, 7% sucrose; #D12450J Research Diets) or Western diet (45% fat, 17% sucrose; #D12451, Research Diets). EFAD mice were maintained on experimental diets for 12 weeks, an exposure period previously established to yield obesity-induced metabolic impairments in *APOE* mice (Arbones-Mainar et al., 2010; Segev et al., 2016). Body weight and food consumption were recorded weekly.

At the end of the treatment period, mice were anesthetized with inhalant isoflurane and transcardially perfused with ice-cold 0.1 M PBS. The brains were rapidly removed and immersion fixed for 48 h in 4% paraformaldehyde/0.1 M PBS, then stored at 4°C in 0.1 M PBS/0.3% NaN_3_ until processed for immunohistochemistry. Gonadal and retroperitoneal fat pads were dissected and weighed as a measure of adiposity, and snap frozen for RNA extraction. All animal procedures were conducted under protocols approved by the University of Southern California Institutional Animal Care and Use Committee and in accordance with National Institute of Health standards.

### Glucose, cholesterol, and triglyceride measurements

Blood glucose readings were measured after overnight fasting (16 h) every four weeks beginning at week 0 of the 12-week treatment period. Blood was collected from the lateral tail vein and immediately assessed for glucose levels using the Precision Xtra Blood Glucose and Ketone Monitoring System (Abbott Diabetes Care).

Glucose tolerance testing (GTT) was performed at week 11. Fasting, baseline glucose readings were taken after which mice were administered a glucose bolus (2 g/kg body weight) via oral gavage. Blood glucose levels were recorded 15, 30, 60, and 120 min after the glucose bolus was given. Area under the curve (AUC) was calculated.

Plasma cholesterol and triglyceride levels were enzymatically determined at the conclusion of the experiment using commercially available kits (LabAssay Triglycerides #290-63701, Wako Chemicals; Total Cholesterol Colorimetric Assay kit, #K603, BioVision). All samples were run in duplicate according to manufacturer’s instructions.

### Thioflavin-S (Thio-S) staining and quantification

Fixed hemi-brains were fully sectioned in the horizontal plane at 40 μm using a vibratome (Leica Biosystems). Every eighth section was stained for Thio-S (#230456, Sigma-Aldrich) using standard methodology. Sections were mounted and allowed to dry overnight, after which they were washed three times in 50% ethanol for 5 min each, then washed in double-distilled H_2_O before being incubated for 10 min in 1% Thio-S dissolved in H_2_O. Stained slides were then rinsed in 70% ethanol before being dehydrated and coverslipped in aqueous anti-fade mounting medium (Vector Laboratories). Digital images were captured at 20× magnification using an Olympus BX50 microscope equipped with a DP74 camera and CellSens software (Olympus). The number of spherical thioflavin-positive deposits were counted using NIH ImageJ 1.50i (United States National Institutes of Health) with the cell counter plugin to mark stained plaque-like structures. Thioflavin-positive deposits were counted in entorhinal cortex (three fields/section), subiculum (two fields/section), and hippocampal subfields CA1 (three fields/section) and CA2/3 (three fields/section), across four sections per animal, for a total of ∼44 fields per brain.

### Immunohistochemistry

Immunohistochemistry was performed using a standard avidin/biotin peroxidase approach with ABC Vector Elite kits (Vector Laboratories). Aβ immunohistochemistry was performed on every eighth section using sections immediately adjacent to those processed for Thio-S. Briefly, sections were pretreated with 95% formic acid for 5 min, then rinsed in TBS before being treated with an endogenous peroxidase blocking solution for 10 min. After three 10 min washes in 0.1% Triton-X/TBS, sections were incubated for 30 min in a blocking solution consisting of 2% bovine serum albumin in TBS. Blocked sections were incubated overnight at 4°C in primary antibody directed against Aβ (#71-5800, 1:300 dilution, Invitrogen) that was diluted in blocking solution. Next, sections were rinsed and incubated in biotinylated secondary antibody diluted in blocking solution. Immunoreactivity was visualized using 3,3’-diaminobenzidine (Vector Laboratories). Additional sections were similarly immunostained without formic acid pretreatment using IBA-1 (#019-19741, 1:2000 dilution, Wako) and GFAP (#ab7260, 1:1000 dilution, Abcam).

To quantify the percentage area occupied by Aβ immunoreactivity (Aβ load), images of nonoverlapping fields were taken at 20× magnification in entorhinal cortex (three fields/section), subiculum (three fields/section), and hippocampal subfields CA1 (five fields/section) and CA2/3 (three fields/section) across 4 tissue sections, for a total of ∼56 images per brain. Images were digitally captured using an Olympus BX50 microscope and DP74 camera paired with a computer running CellSens software (Olympus). The pictures were converted to grayscale images and thresholded using NIH ImageJ 1.50i to yield binary images separating positive and negative immunostaining. Aβ load was calculated as the percentage of the total area that was positively immunolabeled.

Microglia and astrocyte activation was quantified using live imaging (Olympus BX50, CASTGrid software, Olympus) at 40× magnification. Each cell was categorized as either resting or reactive based on its morphology, as reported in previous studies (Ayoub and Salm, 2003; Wilhelmsson et al., 2006). Specifically, microglia were scored as resting (type 1) if they had spherical cell bodies, with numerous thin, highly ramified processes. Cells were scored as type 2 cells if they exhibited enlarged rod-shaped cell bodies with fewer processes that were shorter and thicker, and scored type 3 cells if they had very few or no processes or several filopodial processes. Both type 2 and type 3 morphologies were considered an activated microglia phenotype. Astrocytes were visualized with GFAP immunostaining and categorized as exhibiting either nonreactive (normally sized cell bodies with a few rather short projections) or reactive (both cell bodies and projections are enlarged) morphology phenotypes. Entorhinal cortex (four fields/section), subiculum (four fields/section), and hippocampal subfields CA-1 (five fields/section) and CA-2/3 (three fields/section) were quantified for both microglia and astrocytes. The number of cells across brain regions scored for each animal averaged ∼700 microglia and ∼600 astrocytes.

#### RNA isolation and real-time PCR

For RNA extractions, gonadal fat pads and hippocampi were homogenized using TRIzol reagent (Invitrogen), following the manufacturer’s protocol. The RNA pellet was treated with RNase-free DNase I (Epicentre) for 30 min at 37°C, and a phenol/chloroform extraction was performed to isolate RNA. The iScript cDNA synthesis system (Bio-Rad) was used to reverse transcribe cDNA from 1 μg of purified RNA. Real-time quantitative PCR was performed on the resulting cDNA using SsoAdvanced Universal SYBR Green Supermix (Bio-Rad) and a Bio-Rad CFX Connect Thermocycler. All measurements were performed in duplicates. Quantification of PCR products was conducted by normalizing with a combination of corresponding hypoxanthine-guanine phosphoribosyltransferase (HPRT) and succinate dehydrogenase [ubiquinone] flavoprotein subunit, mitochondrial (SDHA) expression levels from the gonadal fat samples, and with β-actin expression levels from hippocampus, using the ΔΔ-CT method to obtain relative mRNA levels. Gonadal fat was probed for levels of cluster of differentiation factor 68 (CD68) and EGF-like module-containing mucin-like hormone receptor-like 1 (F4/80), while hippocampus was probed for β-secretase 1 (BACE1), neprilysin, insulin-degrading enzyme (IDE), CD68, glial fibrillary acidic protein (GFAP), and cluster of differentiation factor 74 (CD74). Primer pair sequences are shown in Table 1.

**Table 1.** Gene targets for the PCR analyses are listed with their corresponding oligonucleotide sequences for the forward and reverse primers

Target gene	Sequence
CD68	Forward: 5’-TTCTGCTGTGGAAATGCAAG-3’ Reverse: 5’-AGAGGGGCTGGTAGGTTGAT-3’
F4/80	Forward: 5’-TGCATCTAGCAATGGACAGC-3’ Reverse: 5’-GCCTTCTGGATCCATTTGAA-3’
HPRT	Forward: 5’-AAGCTTGCTGGTGAAAAGGA-3’ Reverse: 5’-TTGCGCTCATCTTAGGCTTT-3’
SDHA	Forward: 5’-ACACAGACCTGGTGGAGACC-3’ Reverse: 5’-GGATGGGCTTGGAGTAATCA-3’
Neprilysin	Forward: 5’-GAGAAAAGCCCACTTGCTTG-3’ Reverse: 5’-GAAAGACAAAATGGGGCAGA-3’
BACE1	Forward: 5’-TCGCTGTCTCACAGTCATCC-3’ Reverse: 5’-AACAAACGGACCTTCCACTG-3’
IDE	Forward: 5’-TGTTTCCACACACAGGCAAT-3’ Reverse: 5’-ACCTGTGAAAAGCCGAGAGA-3’
CD74	Forward: 5’-CAAGTACGGCAACATGACCC-3’ Reverse: 5’-GCACTTGGTCAGTACTTTAGGTG-3’
GFAP	Forward: 5’-AACGACTATCGCCGCCAACTG-3’ Reverse: 5’-CTCTTCCTGTTCGCGCATTTG-3’
β-Actin	Forward: 5’-AGCCATGTACGTAGCCATCC-3’ Reverse: 5’-CTCTCAGCTGTGGTGGTGAA-3’

### Statistical analyses

For the analysis of body weight and glucose tolerance data, two-way repeated measures ANOVAs were run using the Statistical Package for Social Sciences (SPSS; version 23, IBM). All other data were analyzed by two-way ANOVA using Prism (version 5, GraphPad Software). In the case of significant main effects, planned comparisons between groups of interest were made using the Bonferroni correction. All data are presented as the mean ± SEM. Significance was set at a threshold of *p* < 0.05. Statistical results are presented in Tables 2, 3.

**Table 2.** Statistical table

Figure	Kolmogorov-Smirnov test for normality (*p* value)	Statistical significance
Figure 1*A* body weight	All groups at all time points are normally distributed (*p* > 0.05).	Genotype: *F*_(1,29)_ = 0.10, *p* = 0.759 diet: *F*_(1,29)_ = 10.51, *p* = 0.003 interaction: *F*_(1,29)_ = 2.68, *p* = 0.112
Figure 1*B* plasma cholesterol	E3FAD CTL > 0.10 E3FAD WD > 0.10 E4FAD CTL > 0.10 E4FAD WD > 0.10	Genotype: *F*_(1,29)_ = 2.86, *p* = 0.103 diet: *F*_(1,29)_ = 1.58, *p* = 0.221 interaction: *F*_(1,29)_ = 2.60, *p* = 0.119
Figure 1*C* plasma triglycerides	E3FAD CTL > 0.10 E3FAD WD > 0.10 E4FAD CTL > 0.10 E4FAD WD > 0.10	Genotype: *F*_(1,29)_ = 0.56, *p* = 0.46 diet: *F*_(1,29)_ = 2.87, *p* = 0.102 interaction: *F*_(1,29)_ = 1.91, *p* = 0.179
Figure 1*D* gonadal fat weight	E3FAD CTL > 0.10 E3FAD WD > 0.10 E4FAD CTL > 0.10 E4FAD WD > 0.10	Genotype: *F*_(1,29)_ = 0.18, *p* = 0.673 diet: *F*_(1,29)_ = 37.04, *p* < 0.001 interaction: *F*_(1,29)_ = 5.01, *p* = 0.033
Figure 1*E* CD68	E3FAD CTL N/A E3FAD WD > 0.10 E4FAD CTL = 0.004 E4FAD WD > 0.10	Genotype: *F*_(1,21)_ = 0.90, *p* = 0.353 diet: *F*_(1,21)_ = 11.54, *p* = 0.003 interaction: *F*_(1,21)_ = 0.85, *p* = 0.366
Figure 1*F* F4/80	E3FAD CTL N/A E3FAD WD > 0.10 E4FAD CTL > 0.10 E4FAD WD > 0.10	Genotype: *F*_(1,21)_ = .09, *p* = .768 diet: *F*_(1,21)_ = 7.02, *p* = 0.015 interaction: *F*_(1,21)_ = 1.19, *p* = 0.288
Figure 1*G* glucose (GTT)	All groups at all time points are normally distributed (*p* > 0.05), except: E4FAD CTL 0 min = 0.002 E4FADWD 15 min = 0.025 E3FAD WD 30 min = 0.011 E4FAD WD 30 min = 0.008	Genotype: *F*_(1,29)_ = 0.02, *p* = 0.886 diet: *F*_(1,29)_ = 5.03, *p* = 0.033 interaction: *F*_(1,29)_ = 0.10, *p* = 0.750
Figure 1*H* GTT AUC	E3FAD CTL = 0.07 E3FAD WD = 0.097 E4FAD CTL > 0.10 E4FAD WD = 0.033	Genotype: *F*_(1,29)_ = .06, *p* = 0.817 diet: *F*_(1,29)_ = 5.73, *p* = 0.023 interaction: *F*_(1,29)_ = 0.12, *p* = 0.737
Figure 1*I* percent glucose change	E3FAD CTL > 0.10 E3FAD WD > 0.10 E4FAD CTL > 0.10 E4FAD WD > 0.10	Genotype: *F*_(1,29)_ = .83, *p* = 0.371 diet: *F*_(1,29)_ = 3.84, *p* = 0.059 interaction: *F*_(1,29)_ = 0.90, *p* = 0.352
Figure 2B Thio-S: entorhinal cortex	E3FAD CTL > 0.10 E3FAD WD = 0.049 E4FAD CTL > 0.10 E4FAD WD > 0.10	Genotype: *F*_(1,29)_ = 50.30, *p* < 0.001 diet: *F*_(1,29)_ = 6.62, *p* = 0.016 interaction: *F*_(1,29)_ = 4.09, *p* = 0.053
Figure 2C Thio-S: subiculum	E3FAD CTL > 0.10 E3FAD WD > 0.10 E4FAD CTL > 0.10 E4FAD WD > 0.10	Genotype: *F*_(1,29)_ = 59.40, *p* < 0.001 diet: *F*_(1,29)_ = 2.98, *p* = 0.095 interaction: *F*_(1,29)_ = 9.75, *p* = 0.004
Figure 2D Thio-S: CA1	E3FAD CTL > 0.10 E3FAD WD > 0.10 E4FAD CTL > 0.10 E4FAD WD > 0.10	Genotype: *F*_(1,29)_ = 80.58, *p* < 0.001 diet: *F*_(1,29)_ = 4.95, *p* = 0.034 interaction: *F*_(1,29)_ = 8.41, *p* = 0.007
Figure 2E Thio-S: CA2/3	E3FAD CTL > 0.10 E3FAD WD > 0.10 E4FAD CTL > 0.10 E4FAD WD > 0.10	Genotype *F*_(1,29)_ = 46.39, *p* < 0.001 diet: *F*_(1,29)_ = 7.41, *p* = 0.011 interaction: *F*_(1,29)_ = 7.32, *p* = 0.011
Figure 3B Aβ load: entorhinal cortex	E3FAD CTL > 0.10 E3FAD WD = 0.002 E4FAD CTL > 0.10 E4FAD WD > 0.10	Genotype *F*_(1,29)_ = 21.38, *p* < 0.001 diet: *F*_(1,29)_ = 7.83, *p* = 0.009 interaction: *F*_(1,29)_ = 4.91, *p* = 0.035
Figure 3C Aβ load: subiculum	E3FAD CTL > 0.10 E3FAD WD > 0.10 E4FAD CTL > 0.10 E4FAD WD > 0.10	Genotype *F*_(1,29)_ = 25.40, *p* < 0.001 diet: *F*_(1,29)_ = 11.19, *p* = 0.002 interaction: *F*_(1,29)_ = 0.11, *p* = 0.742
Figure 3D Aβ load: CA1	E3FAD CTL > 0.10 E3FAD WD > 0.10 E4FAD CTL > 0.10 E4FAD WD = 0.036	Genotype *F*_(1,29)_ = 37.66, *p* < 0.001 diet: *F*_(1,29)_ = 2.91, *p* = 0.099 interaction: *F*_(1,29)_ = 2.71, *p* = 0.110
Figure 3E Aβ load: CA2/3	E3FAD CTL > 0.10 E3FAD WD > 0.10 E4FAD CTL > 0.10 E4FAD WD > 0.10	Genotype *F*_(1,29)_ = 47.27, *p* < 0.001 diet: *F*_(1,29)_ = 10.36, *p* = 0.003 interaction: *F*_(1,29)_ = 4.48, *p* = 0.043
Figure 4B microglia number: entorhinal cortex	E3FAD CTL > 0.10 E3FAD WD > 0.10 E4FAD CTL > 0.10 E4FAD WD > 0.10	Genotype *F*_(1,27)_ = 9.78, *p* = 0.004 diet: *F*_(1,27)_ = 2.31, *p* = 0.141 interaction: *F*_(1,27)_ = 1.05, *p* = 0.316
Figure 4C microglia number: subiculum	E3FAD CTL > 0.10 E3FAD WD > 0.10 E4FAD CTL > 0.10 E4FAD WD > 0.10	Genotype *F*_(1,27)_ = 42.77, *p* < 0.001 diet: *F*_(1,27)_ = 4.20, *p* = 0.050 interaction: *F*_(1,27)_ = 4.75, *p* = 0.038
Figure 4D microglia number: CA1	E3FAD CTL > 0.10 E3FAD WD > 0.10 E4FAD CTL > 0.10 E4FAD WD > 0.10	Genotype *F*_(1,27)_ = 51.42, *p* < 0.001 diet: *F*_(1,27)_ = 10.78, *p* = 0.003 interaction: *F*_(1,27)_ = 7.97, *p* = 0.009
Figure 4E microglia number: CA2/3	E3FAD CTL > 0.10 E3FAD WD > 0.10 E4FAD CTL > 0.10 E4FAD WD > 0.10	Genotype *F*_(1,27)_ = 21.64, *p* < 0.001 diet: *F*_(1,27)_ = 1.97, *p* = 0.172 interaction: *F*_(1,27)_ = 1.90, *p* = 0.180
Figure 4F microglia reactivity: entorhinal cortex	E3FAD CTL > 0.10 E3FAD WD > 0.10 E4FAD CTL > 0.10 E4FAD WD > 0.10	Genotype *F*_(1,27)_ = 109.10, *p* < 0.001 diet: *F*_(1,27)_ = 1.64, *p* = 0.212 interaction: *F*_(1,27)_ = 5.52, *p* = 0.027
Figure 4G microglial reactivity: subiculum	E3FAD CTL > 0.10 E3FAD WD > 0.10 E4FAD CTL = 0.07 E4FAD WD < 0.001	Genotype *F*_(1,27)_ = 19.70, *p* < 0.001 diet: *F*_(1,27)_ = 0.00, *p* = 0.995 interaction: *F*_(1,27)_ = 0.51, *p* = 0.480
Figure 4H microglial reactivity: CA1	E3FAD CTL > 0.10 E3FAD WD > 0.10 E4FAD CTL > 0.10 E4FAD WD = 0.04	Genotype *F*_(1,27)_ = 78.70, *p* < 0.001 diet: *F*_(1,27)_ = 5.00, *p* = 0.034 interaction: *F*_(1,27)_ = 11.58, *p* = 0.002
Figure 4I microglial reactivity: CA2/3	E3FAD CTL > 0.10 E3FAD WD > 0.10 E4FAD CTL > 0.10 E4FAD WD > 0.10	Genotype *F*_(1,27)_ = 165.70, *p* < 0.001 diet: *F*_(1,27)_ = 21.04, *p* < 0.001 interaction: *F*_(1,27)_ = 32.66, *p* < 0.001
Figure 5B astrocyte number: entorhinal cortex	E3FAD CTL > 0.10 E3FAD WD > 0.10 E4FAD CTL > 0.10 E4FAD WD > 0.10	Genotype *F*_(1,29)_ = 3.82, *p* = 0.060 diet: *F*_(1,29)_ = 0.29, *p* = 0.593 interaction: *F*_(1,29)_ = 0.41, *p* = 0.528
Figure 5C astrocyte number: subiculum	E3FAD CTL > 0.10 E3FAD WD > 0.10 E4FAD CTL > 0.10 E4FAD WD > 0.10	Genotype *F*_(1,29)_ = 9.95, *p* = 0.004 diet: *F*_(1,29)_ = 4.79, *p* = 0.037 interaction: *F*_(1,29)_ = 1.04, *p* = 0.316
Figure 5D astrocyte number: CA1	E3FAD CTL > 0.10 E3FAD WD > 0.10 E4FAD CTL > 0.10 E4FAD WD > 0.10	Genotype *F*_(1,29)_ = 5.88, *p* = 0.022 diet: *F*_(1,29)_ = 3.55, *p* = 0.069 interaction: *F*_(1,29)_ = 0.49, *p* = 0.489
Figure 5E astrocyte number: CA2/3	E3FAD CTL > 0.10 E3FAD WD > 0.10 E4FAD CTL > 0.10 E4FAD WD > 0.10	Genotype *F*_(1,29)_ = 1.82, *p* = 0.188 diet: *F*_(1,29)_ = 4.26, *p* = 0.048 interaction: *F*_(1,29)_ = 0.02, *p* = 0.894
Figure 5F astrocyte reactivity: entorhinal cortex	E3FAD CTL > 0.10 E3FAD WD = 0.004 E4FAD CTL > 0.10 E4FAD WD > 0.10	Genotype *F*_(1,29)_ = 46.97, *p* < 0.001 diet: *F*_(1,29)_ = 5.75, *p* = 0.023 interaction: *F*_(1,29)_ = 4.82, *p* = 0.036
Figure 5G astrocyte reactivity: subiculum	E3FAD CTL > 0.10 E3FAD WD > 0.10 E4FAD CTL = 0.045 E4FAD WD > 0.10	Genotype *F*_(1,29)_ = 27.72, *p* < 0.001 diet: *F*_(1,29)_ = 3.13, *p* = 0.088 interaction: *F*_(1,29)_ = 0.00, *p* = 0.989
Figure 5H astrocyte reactivity: CA1	E3FAD CTL > 0.10 E3FAD WD > 0.10 E4FAD CTL > 0.10 E4FAD WD > 0.10	Genotype *F*_(1,29)_ = 87.49, *p* < 0.001 diet: *F*_(1,29)_ = 23.82, *p* < 0.001 interaction: *F*_(1,29)_ = 2.08, *p* = 0.160
Figure 5I astrocyte reactivity: CA2/3	E3FAD CTL > 0.10 E3FAD WD > 0.10 E4FAD CTL > 0.10 E4FAD WD > 0.10	Genotype *F*_(1,29)_ = 11.68, *p* = 0.002 diet: *F*_(1,29)_ = 7.83, *p* = 0.009 interaction: *F*_(1,29)_ = 2.405, *p* = 0.132

**Table 3.** Relative gene expression in hippocampus

Gene	Mean ± SEM	Kolmogorov-Smirnov test for normality (*p* value)	Statistical significance
BACE1	E3FAD CTL = 1 ± N/A E3FAD WD = 1.53 ± 0.31 E4FAD CTL = 1.32 ± 0.19 E4FAD WD = 1.76 ± 0.41	E3FAD CTL > 0.10 E3FAD WD > 0.10 E4FAD CTL > 0.10 E4FAD WD > 0.10	Genotype: *F*_(1,28)_ = 1.10, *p* = 0.304 diet: *F*_(1,28)_ = 3.44, *p* = 0.074 interaction: *F*_(1,28)_ = 0.03, *p* = 0.874
Neprilysin	E3FAD CTL = 1 ± N/A E3FAD WD = 1.61 ± 0.79 E4FAD CTL = 0.94 ± 0.30 E4FAD WD = 1.79 ± 0.63	E3FAD CTL > 0.10 E3FAD WD > 0.10 E4FAD CTL > 0.10 E4FAD WD > 0.10	Genotype: *F*_(1,28)_ = 0.02, *p* = 0.902 diet: *F*_(1,28)_ = 2.49, *p* = 0.126 interaction: *F*_(1,28)_ = 0.06, *p* = 0.802
IDE	E3FAD CTL = 1 ± N/A E3FAD WD = 1.27 ± 0.39 E4FAD CTL = 1.30 ± 0.39 E4FAD WD = 1.12 ± 0.35	E3FAD CTL > 0.10 E3FAD WD > 0.10 E4FAD CTL = 0.01 E4FAD WD > 0.10	Genotype: *F*_(1,28)_ = 0.08, *p* = 0.785 diet: *F*_(1,28)_ = 0.00, *p* = 0.955 interaction: *F*_(1,28)_ = 0.49, *p* = 0.489
CD68	E3FAD CTL = 1 ± N/A E3FAD WD = 1.21 ± 0.29 E4FAD CTL = 1.74 ± 0.30 E4FAD WD = 2.30 ± 0.29	E3FAD CTL > 0.10 E3FAD WD > 0.10 E4FAD CTL > 0.10 E4FAD WD > 0.10	Genotype: *F*_(1,28)_ = 10.75, *p* = 0.003 diet: *F*_(1,28)_ = 1.91, *p* = 0.178 interaction: *F*_(1,28)_ = 0.40, *p* = 0.532
GFAP	E3FAD CTL = 1 ± N/A E3FAD WD = 1.02 ± 0.11 E4FAD CTL = 1.56 ± 0.21 E4FAD WD = 2.70 ± 0.04	E3FAD CTL > 0.10 E3FAD WD > 0.10 E4FAD CTL > 0.10 E4FAD WD > 0.10	Genotype: *F*_(1,28)_ = 14.26, *p* < 0.001 diet: *F*_(1,28)_ = 0.23, *p* = 0.634 interaction: *F*_(1,28)_ = 0.14, *p* = 0.712
CD74	E3FAD CTL = 1 ± N/A E3FAD WD = 1.28 ± 0.28 E4FAD CTL = 3.32 ± 0.62 E4FAD WD = 5.04 ± 1.30	E3FAD CTL > 0.10 E3FAD WD = 0.01 E4FAD CTL > 0.10 E4FAD WD > 0.10	Genotype: *F*_(1,28)_ = 16.98, *p* < 0.001 diet: *F*_(1,28)_ = 1.86, *p* = 0.184 interaction: *F*_(1,28)_ = 0.96, *p* = 0.335

Data are presented as mean fold differences (±SEM) relative to E3FAD mice on a control diet. The Kolmogorov-Smirnov test for normality was performed, with *p* > 0.05 indicating a normal distribution. Genes related to β-amyloid production (BACE-1) and clearance (neprilysin, IDE) showed no significant changes with either diet or genotype, while genes related to glial activation (CD68, GFAP, and CD74) were increased in E4FAD mice on both control and Western diets.

## Results

### Obesity-related outcomes of Western diet

**Figure 1.**
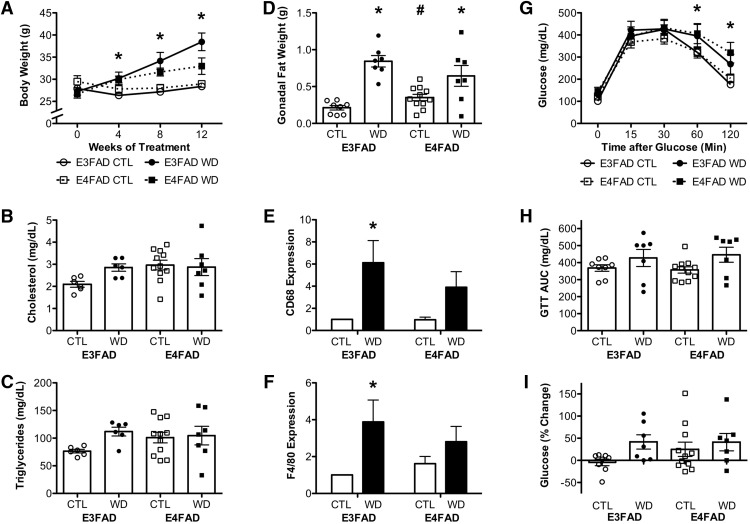
Metabolic outcomes associated with DIO in E3FAD and E4FAD mice. ***A***, Body weights in male E3FAD and E4FAD mice maintained on control (CTL) and Western (WD) diets taken at baseline (week 0) and four-week intervals across the 12-week experimental period. Plasma levels of cholesterol (***B***) and triglyceride levels (***C***) in E3FAD and E4FAD mice on control and Western diets at the end of the experimental period. ***D***, Weight of the gonadal fat pads across groups. Relative mRNA expression of macrophage markers (***E***) CD68 and (***F***) F4/80 in gonadal fat, as determined by real time PCR. Data show fold differences relative to the E3FAD + control diet group. ***G***, GTT showing blood glucose levels over time after a glucose bolus. ***H***, AUC for the GTT. ***I***, Percentage change in fasting blood glucose levels relative to baseline after 12-weeks of control or Western diet. Data are presented as mean (±SEM) values; *n* = 7–11/group. E3FAD mice are shown as circles, E4FAD mice are shown as squares; control diet groups are indicated as open symbols or bars, whereas Western diet groups are filled symbols or bars. *, *p* < 0.05 relative to genotype-matched mice in control diet condition. #, *p* < 0.05 relative to E3FAD mice in same diet condition.

To begin investigating whether there are gene X environment interactions between *APOE* and Western diet, we first compared measures of DIO in E3FAD versus E4FAD mice following the 12-week exposure to control and Western diets. The control diet was associated with <1% gain in body weight in both E3FAD and E4FAD mice, whereas Western diet yielded a 39 ± 7.7% increase in body weight in E3FAD and a 24 ± 7.21% increase in E4FAD mice (Fig. 1*A*), such that the effects of diet did not vary significantly across genotypes (*p* = 0.112; Fig. 1*A*; Table 2). A 2 × 2 repeated measures ANOVA revealed a significant main effect of diet on body weight (*F* = 10.51, *p* = 0.003; Fig. 1*A*) in which Western diet was associated with increased weight. *APOE* genotype did not significantly affect body weight (*p* = 0.759; Fig. 1*A*). Between group comparisons revealed that E3FAD mice fed a Western diet weighed significantly more than E3FAD mice fed a control diet at 4, 8, and 12 weeks (*p* < 0.05). There were no statistically significant differences in body weights at any time point between control and Western diet groups in E4FAD mice.

We next examined plasma levels of cholesterol and triglycerides as measures of adverse effects of Western diet. We found that plasma cholesterol levels were significantly affected by neither genotype (*p* = 0.103) nor diet (*p* = 0.221), and we did not find an interaction effect (*p* = 0.119; Fig. 1*B*; Table 2). Likewise, there were no effects of either genotype (*p* = 0.46) or diet (*p* = 0.102), or an interaction effect (*p* = 0.179) on plasma triglyceride levels (Fig. 1*C*).

Because metabolic impairments associated with obesity have been linked to adiposity, we assessed fat deposition across groups. We observed a significant interaction effect (*F* = 5.01, *p* = 0.033; Table 2), such that on the control diets, E4FAD mice had more gonadal fat than E3FADs (*p* = 0.027), but there was no difference between E3FAD and E4FAD mice on Western diet (*p* = 0.230; Fig. 1*D*). Additionally, there was a significant main effect of diet (*F* = 37.04, *p* < 0.001) on weight of the gonadal fat pads, so that both E3FAD and E4FAD mice had increased fat pads with Western diet (Fig. 1*D*). Parallel findings were observed in the retroperitoneal fat pads (data not shown). Because inflammation is an established hallmark of obesity, we examined gene expression of the macrophage markers CD68 and F4/80 by PCR in the adipose tissue. We found a significant main effect of diet on CD68 expression (*F* = 11.54, *p* = 0.003), although this effect reached statistical significance only in E3FAD but not in E4FAD mice (Fig. 1*E*). There was no statistically significant effect of genotype (*p* = 0.353), nor was there an interaction between diet and genotype (*p* = 0.366) on CD68 expression. Diet had a main effect on adipose F4/80 expression (*F* = 7.02, *p* = 0.015), and again, this effect reached statistical significance only in E3FAD mice (Fig. 1*F*). There was no statistically significant effect of genotype (*p* = .768), and no interaction effect (*p* = 0.288) on F4/80 expression (Table 2).

In addition to increasing body weight and adiposity, Western diet can induce metabolic impairments including dysregulation of glucose homeostasis. When examining glucose clearance in the GTT, we found a significant main effect of diet (*F* = 5.03, *p* = 0.033), such that both E3FAD and E4FAD mice fed a Western diet were impaired at clearing glucose (Fig. 1*G*; Table 2). There was no main effect of genotype (*p* = 0.886), or interaction effect between diet and genotype (*p* = 0.750) on glucose clearance. We also calculated the area under the curve (AUC) for GTT, and found that there was a significant main effect of diet (*F* = 5.73, *p* = 0.023), but not of genotype (*p* = 0.817) on GTT AUC (Fig. 1*H*). However, the effect of diet failed to reach statistical significance when examined separately in E3FAD and E4FAD mice. There was no interaction between genotype and diet on GTT AUC (*p* = 0.737). Changes in fasting glucose levels over the diet treatment period showed a trend toward a main effect of diet (*F* = 3.84, *p* = 0.059; Fig. 1*I*). There was no effect of genotype (*p* = 0.371) nor was there an interaction between diet and genotype (*p* = 0.352) on changes in glucose levels (Table 2).

### Western diet increases β-amyloid deposition in E4FAD but not in E3FAD mice

**Figure 2.**
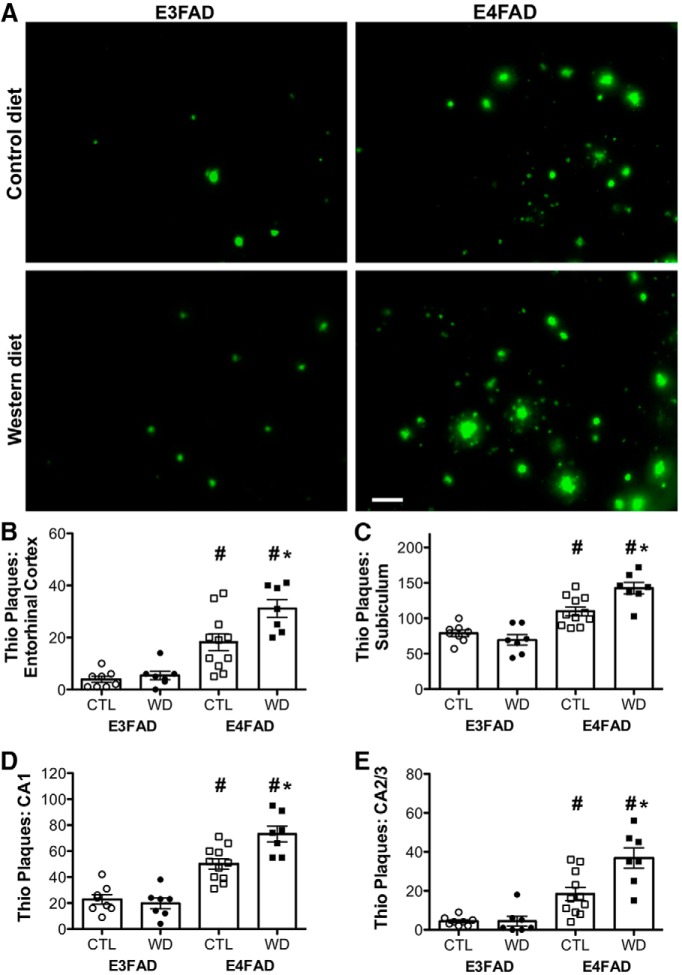
Accumulation of amyloidogenic deposits assessed by Thio-S staining in E3FAD and E4FAD mice across dietary treatments. ***A***, Representative images of Thio-S staining in the subiculum of E3FAD and E4FAD males fed control and Western diets. Scale bar, 50 µm. Numbers of Thio-S positive plaque numbers in E3FAD and E4FAD mice maintained on control and Western diets were quantified in (***B***) entorhinal cortex, and hippocampal subregions (***C***) subiculum, (***D***) CA1, and (***E***) CA2/3. Data are presented as mean (±SEM) values; *n* = 7–11/group. E3FAD mice are shown as circles, E4FAD mice are shown as squares; control diet groups are indicated as open symbols, and Western diet groups as filled symbols. *, *p* < 0.05 relative to genotype-matched mice in control diet condition. #, *p* < 0.05 relative to E3FAD mice in same diet condition.

The primary AD-related neuropathological change in EFAD mice at this age is accumulation of β-amyloid protein, largely in the form of extracellular deposits, many of which exhibit positive Thio-S staining that is indicative of amyloid. Thus, to begin assessing AD-related neuropathology, Thio-S positive plaques were counted in entorhinal cortex and in subregions of the hippocampus. Visual inspection of stained sections qualitatively showed not only the expected increase in amyloid deposits in E4FAD mice, but also the surprising finding that Western diet increased Thio-S positive plaques only in E4FAD mice (Fig. 2*A*). Specifically, there were significant interaction effects between genotype and diet on Thio-S positive plaques in subiculum (*F* = 9.75, *p* = 0.004; Fig. 2*C*), CA1 (*F* = 8.41, *p* = 0.007; Fig. 2*D*), and CA2/3 (*F* = 7.32, *p* = 0.011; Fig. 2*E*), and a nonsignificant trend toward an interaction in entorhinal cortex (*F* = 4.09, *p* = 0.053; Fig. 2*B*; Table 2). Further analyses revealed that diet significantly increased Thio-S positive plaque counts in E4FAD but not E3FAD males across all brain regions sampled (*p* < 0.01). Additionally, there was a significant main effect of genotype even in the absence of diet, such that E4FAD mice had a greater number of Thio-S positive plaques in entorhinal cortex (*F* = 50.30, *p* < 0.001; Fig. 2*B*), subiculum (*F* = 59.40, *p* < 0.001; Fig. 2*C*), CA1 (*F* = 80.58, *p* < 0.001; Fig. 2*D*), and CA2/3 (*F* = 46.39, *p* < 0.001; Fig. 2*E*), than did E3FAD mice.

**Figure 3.**
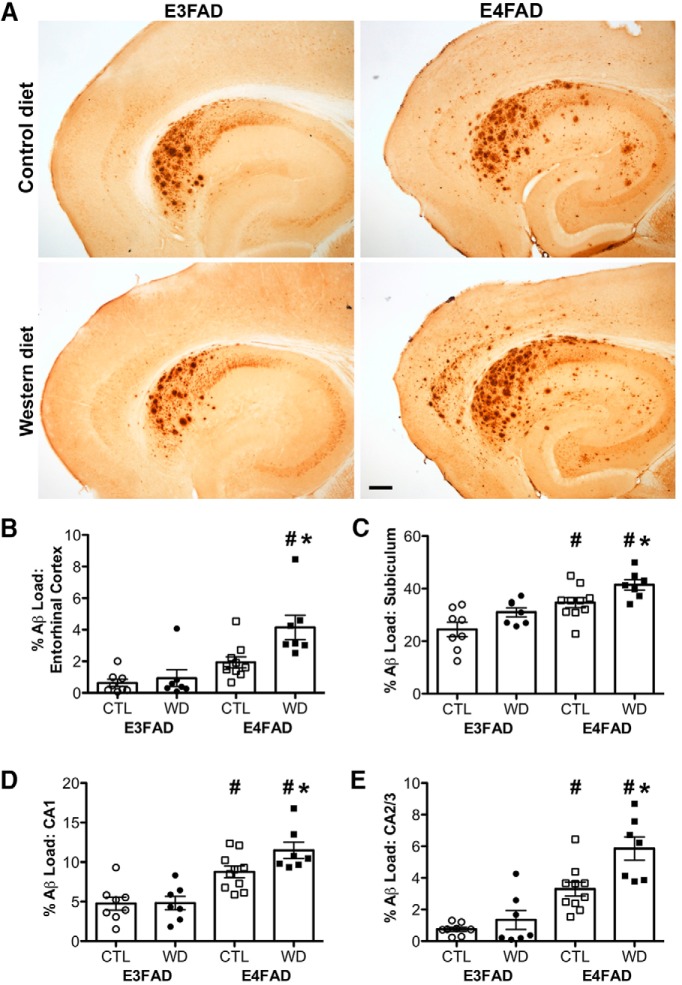
Accumulation of β-amyloid deposits assessed by immunohistochemistry in E3FAD and E4FAD mice across dietary treatments. ***A***, Representative images of β-amyloid immunoreactivity in entorhinal cortex and hippocampus in E3FAD and E4FAD males maintained on control and Western diets. Scale bar, 100 µm. β-Amyloid burden was quantified as immunoreactivity load in E3FAD and E4FAD mice in control and Western diets groups in (***B***) entorhinal cortex, and hippocampal subregions (***C***) subiculum, (***D***) CA1, and (***E***) CA2/3. Data are presented as mean (±SEM) values; *n* = 7–11/group. E3FAD mice are shown as circles, E4FAD mice are shown as squares; control diet groups are indicated as open symbols, and Western diet groups as filled symbols. *, *p* < 0.05 relative to genotype-matched mice in control diet condition. #, *p* < 0.05 relative to E3FAD mice in same diet condition.

As a second measure of AD-like pathology, we assessed total β-amyloid burden by immunohistochemistry. This provides a measure of complete β-amyloid, as the antibody recognizes intra- and extracellular accumulations of Aβ, even those that have not progressed to Thio-S positive amyloid deposits. Results repeated the same general pattern observed with Thio-S staining. That is, (1) E4FAD mice exhibit greater β-amyloid burden, and (2) E4FAD but not E3FAD mice show increased β-amyloid accumulation with Western diet (Fig. 3*A*). We found significant interaction effects between genotype and diet in entorhinal cortex (*F* = 4.91, *p* = 0.035; Fig. 3*B*) and in CA2/3 (*F* = 4.48, *p* = 0.043; Fig. 3*E*), but not in subiculum (*F* = 0.11, *p* = 0.742; Fig. 3*C*) or in CA1 (*F* = 2.71, *p* = 0.110; Fig. 3*D*; Table 2). Bonferroni *post hoc* tests showed that Western diet significantly increased Aβ load in E4FAD but not in E3FAD mice across all brain regions surveyed (*p* < 0.05). There was a significant main effect of genotype with E4FAD mice having greater Aβ load than E3FAD mice in entorhinal cortex (*F* = 21.38, *p* < 0.001; Fig. 3*B*), subiculum (*F* = 25.40, *p* < 0.001; Fig. 3*C*), CA1 (*F* = 37.66, *p* < 0.001; Fig. 3*D*), and CA2/3 (*F* = 47.27, *p* < 0.001; Fig. 3*E*).

### Western diet increases gliosis more strongly in E4FAD than in E3FAD mice

Gliosis is an important neuropathological feature of AD that is also associated with both obesity and *APOE*4. To assess gliosis, we compared both the relative cell numbers and morphologic activation state of microglia and astrocytes across groups. We found that, in comparison to E3FAD mice, E4FAD mice consistently had a higher total number of glial cells as well as a higher percentage of glial cells with reactive versus resting phenotypes. Moreover, the effects of diet on glial number and reactivity were stronger in E4FAD than in E3FAD mice.

**Figure 4.**
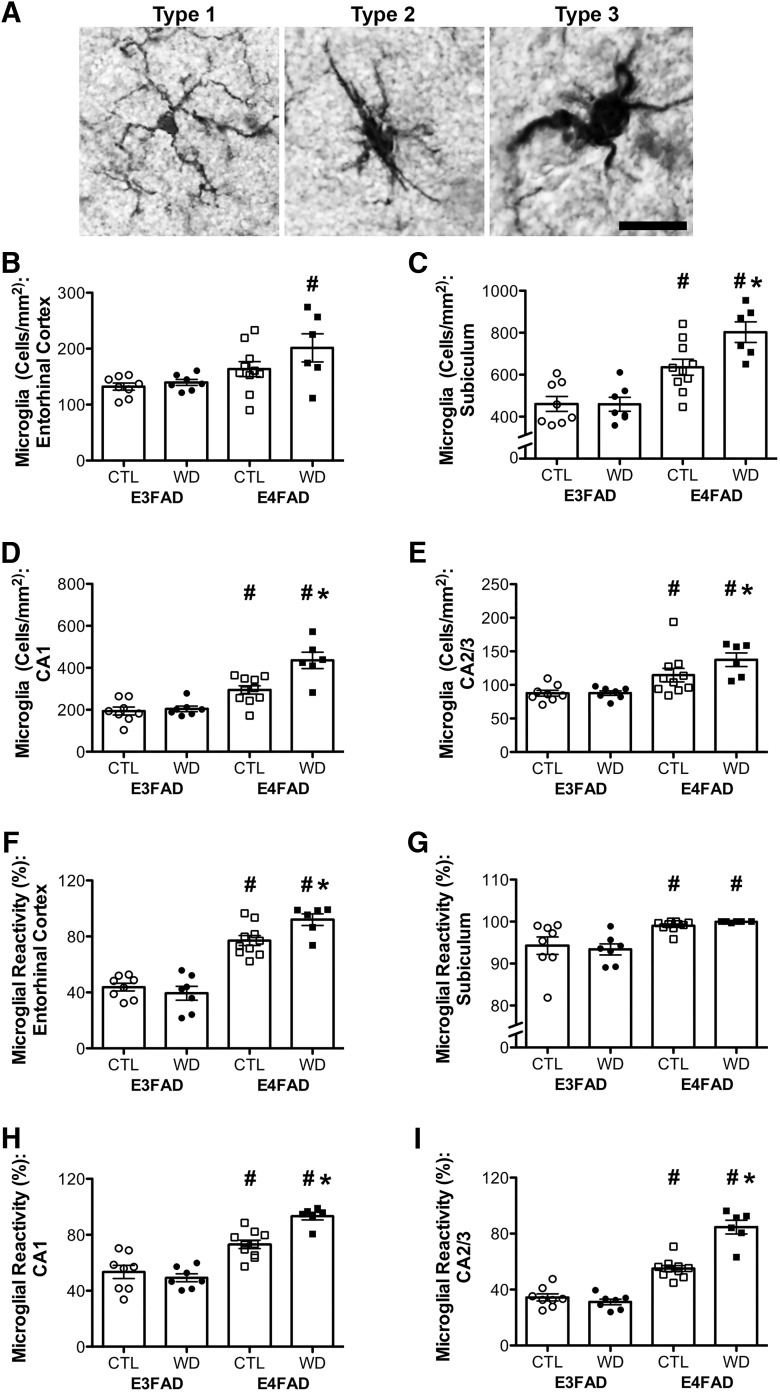
Microglia number and morphologic status assessed by IBA-1 immunohistochemistry in E3FAD and E4FAD mice across dietary treatments. ***A***, Representative images of microglial morphology associated with resting (type 1) and reactive (types 2 and 3) phenotypes. Scale bar, 40 µm. ***B–E***, Densities (cells/mm^2^) of IBA-1-immunoreactive cells in E3FAD and E4FAD mice on control and Western diets were quantified in (***B***) entorhinal cortex, and hippocampal subregions (***C***) subiculum, (***D***) CA1, and **E)** CA2/3. **F–I)** Percentages of all IBA-1-immunoreactive cells scored as having reactive phenotype (types 2 and 3) were quantified in (***F***) entorhinal cortex, and hippocampal subregions (***G***) subiculum, (***H***) CA1, and (***I***) CA2/3. Data are presented as mean (±SEM) values; *n* = 7–11/group. E3FAD mice are shown as circles, E4FAD mice are shown as squares; control diet groups are indicated as open symbols, and Western diet groups as filled symbols. *, *p* < 0.05 relative to genotype-matched mice in control diet condition. #, *p* < 0.05 relative to E3FAD mice in same diet condition.

We first examined microglia number and morphology by IBA-1 staining. Figure 4*A* shows a resting microglial cell with thin, ramified processes (type 1), and activated cells with rod-shaped cell bodies and fewer, thicker processes (type 2), and amoeboid cells (type 3). We found significant interactions between genotype and diet when examining the total number of microglia per mm^2^ in subiculum (*F* = 4.75, *p* = 0.038; Fig. 4*C*) and in CA1 (*F* = 7.97, *p* = 0.009; Fig. 4*D*), with Bonferroni *post hoc* tests showing that Western diet increased microglia number in E4FAD but not in E3FAD mice in these brain regions (*p* < 0.05; Table 2). There were no interaction effects on microglia number in entorhinal cortex (*p* = 0.316; Fig. 4*B*), or in CA2/3 (*p* = 0.180; Fig. 4*E*). There was a significant effect of genotype on the total number of microglia per mm^2^ in entorhinal cortex (*F* = 9.78, *p* = 0.004; Fig. 4*B*), subiculum (*F* = 42.77, *p* < 0.001; Fig. 4*C*), CA1 (*F* = 51.42, *p* < 0.001; Fig. 4*D*), and CA2/3 (*F* = 21.64, *p* < 0.001; Fig 4*E*), such that E4FAD mice had a greater total number of microglia across these brain regions than did E3FAD mice. However, in entorhinal cortex, the effect of genotype was significant only in animals on a Western diet.

Measures of microglial reactivity showed similar results as microglial number. Significant interaction effects between genotype and diet were observed in entorhinal cortex (*F* = 5.52, *p* = 0.027; Fig. 4*F*), CA1 (*F* = 11.58, *p* = 0.002; Fig. 4*H*), and CA2/3 (*F* = 32.66, *p* < 0.001; Fig. 4*I*), but not in subiculum (*p* = 0.480; Fig. 4*G*; Table 2). Bonferroni *post hoc* tests revealed that Western diet increased the percentage of reactive microglia in entorhinal cortex, CA1, and CA2/3 of E4FAD, but not E3FAD, male mice. There was a significant main effect of genotype even in the absence of diet, such that E4FAD mice had a greater percentage of reactive microglia than E3FAD mice in entorhinal cortex (*F* = 109.10, *p* < 0.001; Fig. 4*F*), subiculum (*F* = 19.70, *p* < 0.001; Fig. 4*G*), CA1 (*F* = 78.70, *p* < 0.001; Fig. 4*H*), and CA2/3 (*F* = 165.70, *p* < 0.001; Fig. 4*I*).

**Figure 5.**
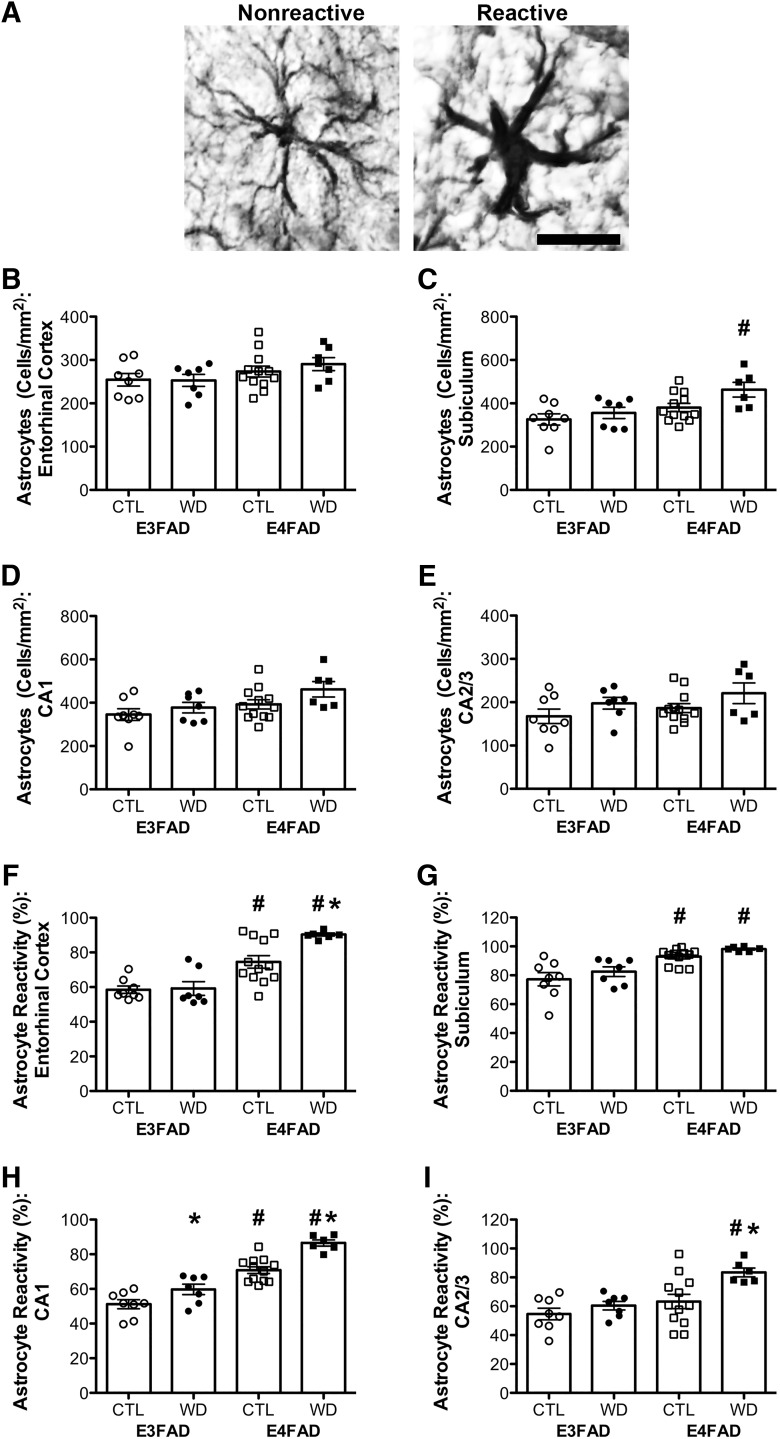
Astrocyte number and morphologic status assessed by GFAP immunohistochemistry in E3FAD and E4FAD mice across dietary treatments. ***A***, Representative images of astrocyte morphology associated with resting and reactive phenotypes. Scale bar, 50 µm. ***B–E***, Densities (cells/mm^2^) of GFAP-immunoreactive cells in E3FAD and E4FAD mice on control and Western diets were quantified in (***B***) entorhinal cortex, and hippocampal subregions (***C***) subiculum, (***D***) CA1, and (***E***) CA2/3. ***F–I***, Percentages of all GFAP-immunoreactive cells scored as having reactive phenotype (type 2) were quantified in (***F***) entorhinal cortex, and hippocampal subregions (***G***) subiculum, (***H***) CA1, and (***I***) CA2/3. Data are presented as mean (±SEM) values; *n* = 7–11/group. E3FAD mice are shown as circles, E4FAD mice are shown as squares; control diet groups are indicated as open symbols, and Western diet groups as filled symbols. *, *p* < 0.05 relative to genotype-matched mice in control diet condition. #, *p* < 0.05 relative to E3FAD mice in same diet condition.

We next examined astrocyte number and activation by GFAP staining. Figure 5*A* shows examples of a nonreactive astrocyte with a normally sized soma versus a reactive phenotype with enlarged soma and projections. For the measure of astrocyte number, the effects of diet did not differ across genotype for any of the brain regions sampled (Table 2). We found significant main effects of genotype on the total number of astrocytes in subiculum (*F* = 9.95, *p* = 0.004; Fig. 5*C*), although this effect was only statistically significant in animals on a Western diet. There was a main effect of genotype on astrocyte number in CA1 (*F* = 5.88, *p* = 0.022; Fig. 5*D*), but this did not reach statistical significance when examined separately in control and Western diet-fed animals. There was a trend toward a significant effect of genotype in entorhinal cortex (*F* = 3.82, *p* = 0.060; Fig. 5*B*), but no effect in CA2/3 (*p* = 0.188; Fig. 5*E*). Diet had significant main effects on astrocyte number in subiculum (*F* = 4.79, *p* = 0.037; Fig. 5*C*), and CA2/3 (*F* = 4.26, *p* = 0.048; Fig. 5*E*), with a trend toward a main effect in CA1 (*F* = 3.55, *p* = 0.069; Fig. 5*D*), although this effect did not reach statistical significance when examined separately in E3FAD and E4FAD mice in any brain region. There was no effect of diet on astrocyte number in entorhinal cortex (*p* = 0.593; Fig. 5*B*).

When examining astrocyte reactivity, we found similar trends as with microglial reactivity. That is, there was a significant interaction effect between genotype and diet on astrocyte reactivity in entorhinal cortex (*F* = 4.82, *p* = 0.036; Fig. 5*F*), with Western diet increasing reactivity only in E4FAD mice (Table 2). There were no significant interaction effects between genotype and diet in subiculum (*p* = 0.989; Fig. 5*G*), CA1 (*p* = 0.160; Fig. 5*H*), or CA2/3 (*p* = 0.132; Fig. 5*I*). Moreover, in the absence of diet, genotype had a significant effect on astrocyte reactivity, with E4FAD mice having a greater percentage of reactive astrocytes in entorhinal cortex (*F* = 46.97, *p* < 0.001; Fig. 5*F*), subiculum (*F* = 27.72, *p* < 0.001; Fig. 5*G*), CA1 (*F* = 87.49, *p* < 0.001; Fig. 5*H*), and CA2/3 (*F* = 11.68, *p* = 0.002; Fig. 5*I*). In CA2/3 the effect of genotype was only significant in Western diet-fed animals. Furthermore, Western diet significantly increased astrocyte reactivity in CA1 (*F* = 23.82, *p* < 0.001; Fig. 5*H*), and CA2/3 (*F* = 7.83, *p* = 0.009; Fig. 5*I*), although this effect was only significant in E4FAD mice in CA2/3. There was a nonsignificant trend toward an effect of diet in subiculum (*F* = 3.13, *p* = 0.088; Fig. 5*G*).

### E4FAD mice have increased gene expression of inflammatory markers

To begin addressing possible mechanisms underlying the interactive effects of *APOE*4 and Western diet, we examined hippocampal gene expression of several markers related to Aβ production and clearance, as well as inflammation. Overall, our results indicate that gene expression of factors involved in Aβ clearance and production are not significantly altered by genotype or diet, and that inflammatory gene expression is increased in E4FAD mice, without being altered by Western diet (Table 3).

For BACE1, relative mRNA levels did not show evidence of an interaction between the diet and *APOE* genotypes (*p* = 0.874). There was no significant main effect genotype (*p* = 0.304), but there was a nonsignificant trend of increased BACE1 levels with Western diet (*p* = 0.074). Expression of the Aβ clearance factor neprilysin was not significantly affected by genotype (*p* = 0.902) or diet (*p* = 0.126), and there was no interaction between genotype and diet (*p* = 0.802). Likewise, gene expression of IDE was not altered by genotype (*p* = 0.785), diet (*p* = 0.955), or the interaction between genotype and diet (*p* = 0.489).

In assessing gene expression of inflammatory markers we found that E4FAD mice had significantly greater levels of the microglial markers CD68 (*F* = 10.75, *p* = 0.003), the astrocyte marker GFAP (*F* = 14.26, *p* < 0.001), and the innate immune marker CD74 (*F* = 16.98, *p* < 0.001), than did E3FAD mice. However, there were no significant effects of diet on levels of CD68 (*p* = 0.178), GFAP (*p* = 0.634), or CD74 (*p* = 0.184). Moreover, there were no significant interactions between genotype and diet on levels of CD68 (*p* = 0.532), GFAP (*p* = 0.712), or CD74 (*p* = 0.335).

## Discussion

The goal of this study is to examine whether *APOE* genotype and obesity interact to promote AD pathogenesis. Comparing E3FAD and E4FAD mice maintained on standard versus Western diets, we demonstrate a significant gene-environment interaction whereby DIO drives AD-related pathology primarily in *APOE*4 mice. Our results are consistent with previous findings in humans (Fitzpatrick et al., 2009; Profenno et al., 2010), and confirm studies in rodent models (Ho et al., 2004; Julien et al., 2010; Kohjima et al., 2010; Barron et al., 2013) that obesity increases risk for development of AD. Similarly, our findings replicate prior rodent data (Fryer et al., 2005; Castellano et al., 2011; Youmans et al., 2012; Rodriguez et al., 2014; Cacciottolo et al., 2016) that model the human observation that *APOE*4 increases the risk and or accelerates the onset of AD pathology (Corder et al., 1993; Saunders et al., 1993; Strittmatter et al., 1993; Morris et al., 2010; Jack et al., 2015). Importantly, our data indicate that the effects of DIO and *APOE*4 are not strictly additive. Although *APOE*4 status is associated with greater AD-like pathology on both control and Western diets, obesity increased AD-like pathology in E4FAD but not E3FAD mice. Our finding that E3FAD mice did not show a diet-induced increase in AD-related pathology is similar to null findings in some rodent models of obesity (Zhang et al., 2013; Knight et al., 2014; Niedowicz et al., 2014), suggesting that deleterious effects of obesity can be regulated by genetic factors besides *APOE4*. Thus, these data suggest an important gene X environment interaction in which *APOE*4 carriers are more susceptible to the AD-promoting effects of obesity.

How neural outcomes in human populations are impacted by the relationship between *APOE* genotype and metabolic risk factors remains incompletely defined. Many studies simply control for *APOE* genotype rather than considering its potential moderating role in the relationship between obesity and AD risk (Vanhanen et al., 2006; Luchsinger et al., 2012). When *APOE* status has been considered as a modulator of AD risk associated with metabolic factors, the results have been mixed. In some studies, *APOE*4 carriers showed significantly more cognitive impairment in association with adverse metabolic conditions including atherosclerosis, peripheral vascular disease, type 2 diabetes (Haan et al., 1999), and high systolic blood pressure at midlife (Peila et al., 2001). Further, levels of senile plaques and neurofibrillary tangles were highest in obese men that were also *APOE*4 carriers (Peila et al., 2002). However, several other studies reported that the AD risk associated with obesity and metabolic syndrome is stronger in *APOE*3 carriers (Dixit et al., 2005; Leiva et al., 2005; Singh et al., 2006; Profenno and Faraone, 2008).

An important consideration in interpreting these seemingly discordant findings is the potential role of sex differences. Although the impact of sex differences in the interactions among obesity, *APOE*, and AD risk has not been thoroughly addressed, AD is characterized by numerous sex differences (Li and Singh, 2014; Pike, 2017). Further, the AD-associated risk of *APOE*4 appears to disproportionately affect women (Payami et al., 1994; Farrer et al., 1997; Altmann et al., 2014). Additionally, there are sex differences in various aspects of obesity (Lovejoy et al., 2009; Mauvais-Jarvis, 2015; Moser and Pike, 2016), including observations that women exhibit relative protection against obesity until menopause (Meyer et al., 2011; Sugiyama and Agellon, 2012; Bloor and Symonds, 2014). Given that sex differences have been found in each of these factors, future studies should address sex as a possible mediator in the relationship between *APOE*4 and obesity. Ongoing projects in our lab have begun to address this issue using female E3FAD and E4FAD mice.

How obesity and *APOE* interact to regulate AD pathogenesis remains to be determined. One candidate mechanism linked to both factors is metabolic impairment. Obesity is strongly associated with development of impaired glucose and insulin metabolism (Kahn et al., 2006; Singla et al., 2010), which are also characteristic of AD patients and have been proposed as possible mechanisms driving AD pathogenesis (Craft, 2005; Martins et al., 2006; Craft, 2009). Notably, *APOE* genotype affects metabolic responses to diet (Snook et al., 1999; Barberger-Gateau et al., 2011), and several studies show that *APOE*4 carriers are at increased risk for a number of metabolic disturbances (de-Andrade et al., 2000; Oh and Barrett-Connor, 2001; Elosua et al., 2003; Marques-Vidal et al., 2003; Sima et al., 2007; Kypreos et al., 2009; Niu et al., 2009; Atabek et al., 2012; Zarkesh et al., 2012; Guan et al., 2013), although some studies find no effect of *APOE* genotype on metabolic outcomes (Meigs et al., 2000; Ragogna et al., 2011). Our findings suggest that E3FAD mice may be more susceptible to some metabolic effects of Western diet, although E4FAD mice trend toward metabolic disturbances even in the absence of a Western diet. Specifically, relative to E4FAD mice, E3FAD mice showed greater diet-induced body weight gain, gonadal fat inflammatory cytokine expression, and higher glucose levels on Western diet. Conversely, E4FAD mice had higher gonadal fat pad weight and a trend toward higher fasting glucose levels than E3FAD mice under the control diet condition. These findings are consistent with several previous reports showing that mice with human *APOE*3 gain more weight in response to a high fat diet than mice with either human *APOE*4 (Arbones-Mainar et al., 2008; Segev et al., 2016) or mouse *APOE* (Karagiannides et al., 2008). It is important to note that the Western diet used in this study has elevated levels of saturated fats, cholesterol, and sucrose, all of which have been independently associated with increased AD-related pathology (Refolo et al., 2000; Oksman et al., 2006; Cao et al., 2007; Takechi et al., 2010). Understanding how *APOE* genotype interacts with various dietary components should be one target of future studies. Though metabolic factors may have a role in AD pathogenesis, our findings that metabolic outcomes of DIO were greater in E3FAD than E4FAD mice argue against the possibility that metabolic impairment significantly contributes to the observed *APOE*4 bias in diet-induced increases in AD-like pathology.

There are several other mechanisms besides metabolic impairment that may contribute to the observed interactions among obesity, *APOE*, and AD-like pathology. One established consequence of obesogenic diets is pro-amyloidogenic alteration in the expression and or activity of factors that regulate generation and clearance of Aβ, including BACE1, neprilysin, and IDE (Standeven et al., 2010; Maesako et al., 2012; Brandimarti et al., 2013; Wei et al., 2014; Maesako et al., 2015). Although we cannot exclude a significant role of such pathways in our observations, we did not observe that mRNA levels of BACE1, neprilysin, and IDE were significantly altered by either the simple or interactive effects of Western diet and *APOE*. Another compelling candidate mechanism is neuroinflammation, which is widely implicated as a significant regulator of AD risk and development of AD pathology (Glass et al., 2010; Wyss-Coray and Rogers, 2012; Heneka et al., 2015). Notably, both obesity and *APOE*4 are associated with increased inflammation in brain and systemically. For example, obesity is linked with increased immune cell infiltration into brain (Buckman et al., 2014), as well as increased glial activation (Koga et al., 2014; Dorfman and Thaler, 2015; Douglass et al., 2017). In addition, obesity increases inflammation in peripheral organs including adipose tissue (Weisberg et al., 2003; Zeyda and Stulnig, 2009) and liver (Park et al., 2010). *APOE*4 is also associated with greater levels of inflammation in the brain (Ophir et al., 2005; Vitek et al., 2009) and throughout the body (Colton et al., 2004; Gale et al., 2014). Moreover, stimulating innate inflammation in the presence of apoE4 increases cell death and damage in macrophages (Cash et al., 2012), and in microglia and neurons (Maezawa et al., 2006a; 2006b). In the context of AD pathology, *APOE*4 is associated with greater glial activation in EFAD mice (Rodriguez et al., 2014). Similarly, we found that both the total number and the relative level of morphologic activation of microglia and astrocytes were higher in E4FAD than E3FAD mice. Further, we observed that E4FAD mice expressed significantly higher mRNA levels of glial markers than E3FAD mice under both control and Western diets. These glial markers were significantly increased across several brain regions in response to DIO in E4FAD but not E3FAD mice. Perhaps in contrast to our results, middle-aged female *APOE*4 mice showed higher levels of neuroinflammation in hippocampus under control diet but decreased neuroinflammation with high-fat diet, relative to age- and sex-matched wild-type mice (Janssen et al., 2016). Though the presence of familial AD transgenes and Aβ pathology in the EFAD model may account for these divergent findings, there may also be age and sex differences in inflammatory responses to both diet and *APOE*4. Further, because reactive astrocytes and microglia are associated with Aβ plaques, the changes in gliosis we observe with *APOE*4 and DIO may be a consequence of, rather than a contributor to, Aβ pathology. Thus, additional research is needed to directly assess the potential mechanistic role of gliosis in the interaction between *APOE*4 and obesity in AD.

To our knowledge, this is the first experimental investigation examining the interaction between *APOE*4 and obesity in the context of AD. Interactions among genetic risk factors like *APOE*4 and environmental and modifiable lifestyle risk factors in AD have thus far not been well studied, although there are some epidemiological studies consistent with this possibility (Dufouil et al., 2000; Hanson et al., 2013; Rajan et al., 2014; Wirth et al., 2014; Ishioka et al., 2016; Zheng and Li, 2016). Our findings suggest that *APOE* genotype affects the relationship between obesity and AD, such that *APOE*4 carriers may be more susceptible to obesity-associated risks than *APOE*3 carriers. This illustrates an important gene-environment interaction and points to the need for additional research exploring such relationships in the context of AD, as well as identifying underlying mechanisms. Additionally, these findings identify a large population that may be at increased risk of AD, but whose chance of developing the disease may be reduced by preventative lifestyle changes.

## Synthesis

Reviewing Editor: Liisa Galea, University of British Columbia Decisions are customarily a result of the Reviewing Editor and the peer reviewers coming together and discussing their recommendations until a consensus is reached. When revisions are invited, a fact-based synthesis statement explaining their decision and outlining what is needed to prepare a revision will be listed below. The following reviewer(s) agreed to reveal their identity: Gregory Cole.

This is a well written manuscript that presents data illustrating interactions between ApoE genotype and the effectiveness of 3 months of a high fat "Western" diet in 3 month old male human ApoE3 or E4 TR (Sullivan) x 5xFAD (Vassar) "EFAD" mice. The Western diet leads to predictable weight gain (obesity) and metabolic (glucose) regulatory changes that are similarly significant with human ApoE3 and ApoE4, but with a selective increase in amyloid plaques and gliosis in the E4FAD mice. The findings are useful in explaining some discrepant results in the literature as there are robust but somewhat inconsistent epidemiological evidence for diet and lifestyle modification of ApoE4 genetic AD risk, the selective effect of high fat Western diet on E4FAD amyloid deposits and gliosis is an important observation from this study. As the authors note, the results are limited to relatively young male mice and the increased gliosis may simply track the amyloid. The reviewers have only a few relatively minor comments and agreed that this will be a nice addition to the literature.

Statistics:

1) A lot of the data are actually not normally distributed, as the authors indicate in Table 1. Although in practice it often makes little difference to the analysis, frequency and ratio variables (i.e., counting plaques and expressing amyloid load as % area) have a Poisson distribution, and are sometimes better treated as nonparametric variables (although these tests are not preferred due to their own limitations). However, normality assumptions can be checked and reported, and this is usually fine when it's done; if not, the data can be converted to an approximately normal distribution (for example, by a logarithmic transformation). A lot of times, this all works out the same way, but sometimes it doesn't. The reason this may be important is that the more interesting outcomes in this paper are interactions, and these in particular are sometimes vulnerable to violations of the underlying ANOVA assumptions. The authors need to at least check these assumptions.

2) Due to the number of significant interactions, it is important to acknowledge these significant interactions first and that when interactions exist it is important to describe these exclusively even in the face of significant main effects. This order is particularly notable for Thio-S results.

3) The results section is very through and perhaps to cut down when there is no significant interaction or main effect the authors could cut out the F and df and simply report the p values if they chose.

4) Bonferroni corrections are very conservative and the authors suggest they use them when analyzing significant main effects (p 8, last line)- but in fact because there are only two groups pairwise comparisons are not needed ( ie 2 genotypes and 2 diets). If Bonferroni were used with significant interactions they authors may consider using Tukey's instead for posthoc comparisons.

5) Were there any effects of WD on APP expression? This would seem important to take into consideration, as this could be the source of changes in amyloid deposition.

6) Were there any differences in peripheral cholesterol in the different mouse lines, either with or without Western Diet?

7) The authors could consider a figure for type 1,2,and 3 morphologies for microglia and astrocytic activation - this could be incorporated into figure 4 or 5.

8) It could be worthwhile to examine PS1 and BACE expression. In many cases of dietary and/or obesity studies we see effects on AD-related pathology, but get little insight into the underlying mechanism. The authors do hint at the problems with establishing mechanism, but don't delve too deeply into the problem. This could also be acknowledged in the discussion.

Discussion

9) The authors point might be helped by incorporating into the discussion more papers that show minimal effects of diet and obesity on amyloid pathology. For example, Niedowicz et al (Acta Neuropathologica Comm, 2014) found no effects on amyloid pathology in a db/db mouse crossed with an APPxPS1 mouse. In another example, Zhang et al (BBA: Mol Basis of Dis, 2013) found little effect of western diet of amyloid in a model of CAA. These papers and others like it - including this one - show that the interactions between metabolism, obesity, and amyloid pathology are more complex than many investigators realize.

10) In this study there is no isolation of the type of fat involved as the diet is high in cholesterol, saturated fat, calories from fat and calories. This invites further study, but does not negate the significance of the main observations and could be added into the discussion on future experiments.

Minor: "APOE4 is associated with greater glial activation EFAD mice (Rodriguez et al., 2014)." Needs "in" EFAD

Minor: Provide a reference for the use of 12 weeks of exposure to the diet.
